# Photo Racemization and Polymerization of (*R*)-1,1′-Bi(2-naphthol)

**DOI:** 10.3390/molecules21111541

**Published:** 2016-11-16

**Authors:** Zhaoming Zhang, Yue Wang, Tamaki Nakano

**Affiliations:** 1 Macromolecular Science Research Division, Institute for Catalysis and Graduate School of Chemical Sciences and Engineering, Hokkaido University, N21 W10, Kita-ku, Sapporo 001-0021, Japan; zhang@polymer.cat.hokudai.ac.jp (Z.Z.); yue.wang@cat.hokudai.ac.jp (Y.W.); 2 Integrated Research Consortium on Chemical Sciences (IRCCS), Institute for Catalysis, Hokkaido University, N21 W10, Kita-ku, Sapporo 001-0021, Japan

**Keywords:** photo-polymerization, racemization, isomerization, excited states, reduction, 1,1′-bi(2-naphthol)

## Abstract

(*R*)-1,1’-Bi(2-naphthol) ((*R*)-BINOL) in an acetonitrile solution lost optical activity upon irradiation with an Hg–Xe lamp. HPLC resolution of the product indicated that (*R*)-BINOL was racemized upon irradiation, and SEC analysis suggested that a polymeric product was formed in the course of racemization. It is proposed that polymerization of BINOL can occur before it is racemized and that a unit in a polymer derived from BINOL may lose its optical activity afterwards due to in-chain racemization and/or reduction. The polymeric products seem to consist not only of BINOL residues but also of residues derived from acetonitrile as well as those derived through reduction of BINOL.

## 1. Introduction

Interactions between substances and light play important roles in life. One of the most important aspects is photosynthesis by plants and other organisms. In photosynthesis, sugars and molecular oxygen are produced from carbon dioxide and water where photon energy drives the reaction. Moreover, in artificial chemical synthesis, light promotes various reactions that are not driven by thermal energy [1,2,3,4,5,6,7,8,9,10,11]. Further, in reactions where chiral compounds are produced, non-racemic products can be obtained using circularly polarized light (CPL) [12,13,14,15,16,17,18,19]. We recently reported that a preferred-handed helical conformation is induced for a polyfluorene derivative in the solid state when the polymer is irradiated with CPL [20,21,22]. The mechanism of this chirality induction involves a twisted-coplanar transition (TCT) of an aromatic–aromatic junction in the polymer where one of the enantiomeric, right- and left-handed twists is preferentially excited into the coplanar conformation. TCT through photo excitation was first predicted for biphenyl through theoretical calculations [23]. In addition, CPL-assisted chirality induction has been reported for polymers consisting of fluorene and azobenzene units [24,25].

**Scheme 1:**
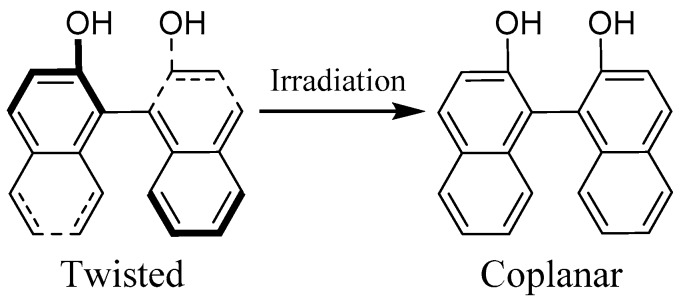
Twisted-coplanar transition (TCT) of **BINOL**.

In this work, in order to examine the applicability and limitations of the photo-induced TCT for a molecule with a higher rotational barrier, we irradiated optically active (*R*)-1,1′-bi(2-naphthol) (BINOL) with non-polarized light and investigated the structures of the products (Scheme 1). BINOL has been used as an effective chiral ligand and as a building block for chiral functional molecules and polymers; its stability has been considered to be sufficient under normal conditions for organic synthesis [26,27,28]. However, the present study indicated that BINOL is readily racemized and polymerized at the same time upon photo irradiation. Such stereo chemical and chemical transformations of BINOL are unprecedented.

## 2. Results and Discussion

**Figure 1:**
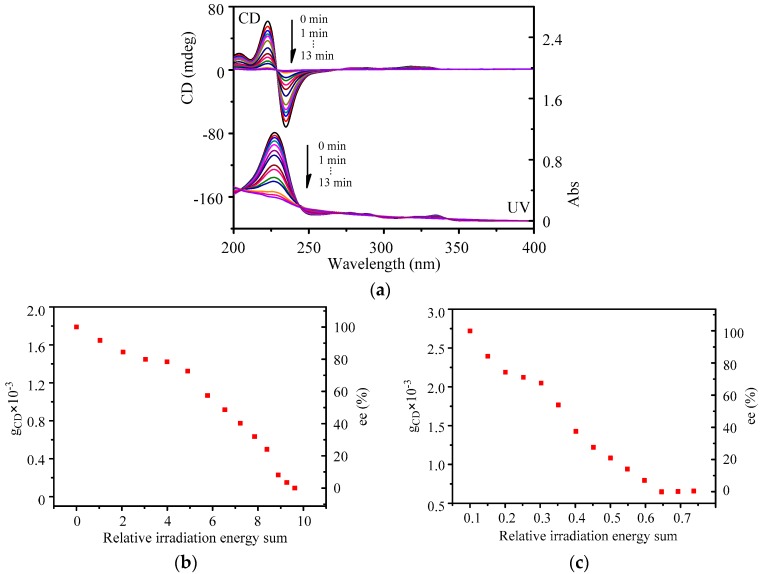
Changes in chirality of (*R*)-BINOL in CH_3_CN solution upon irradiation ([BINOL]_o_ = 1.0 × 10^−4^ M, cell path 1 mm, room temperature): (**a**) CD and UV spectra taken upon irradiation for 13 min at 1-min interval; (**b**) g_CD_-vs.-relative irradiation energy plot at 223 nm; (**c**) g_CD_-vs.-relative irradiation energy plot at 318 nm.

BINOL was irradiated in a solution of acetonitrile under N_2_ atmosphere with light using a 500-W Hg–Xe lamp without any modulation or polarization. General information about experiments is found in Appendix A. A drastic change was observed through irradiation on circular dichroism (CD) spectra in acetonitrile solution (Figure 1a); CD intensity largely decreased through irradiation, and the signal almost disappeared within ca. 15 min of irradiation (Figure 1b,c). In addition, along with the changes in CD spectra, a decrease in UV intensity (hypochromism) was observed. These results are suggestive of stereo chemical and chemical transformation of BINOL through photoexcitation. Hypochromism has been observed in photo chirality induction to poly(9,9-di-*n*-octylflurene-2,7-diyl) using CPL, and it was ascribed to a change in dihedral angle in the main chain [20,21]; however, the hypochromism observed in this work appears to have a connection with polymer formation as discussed later.

**Figure 2:**
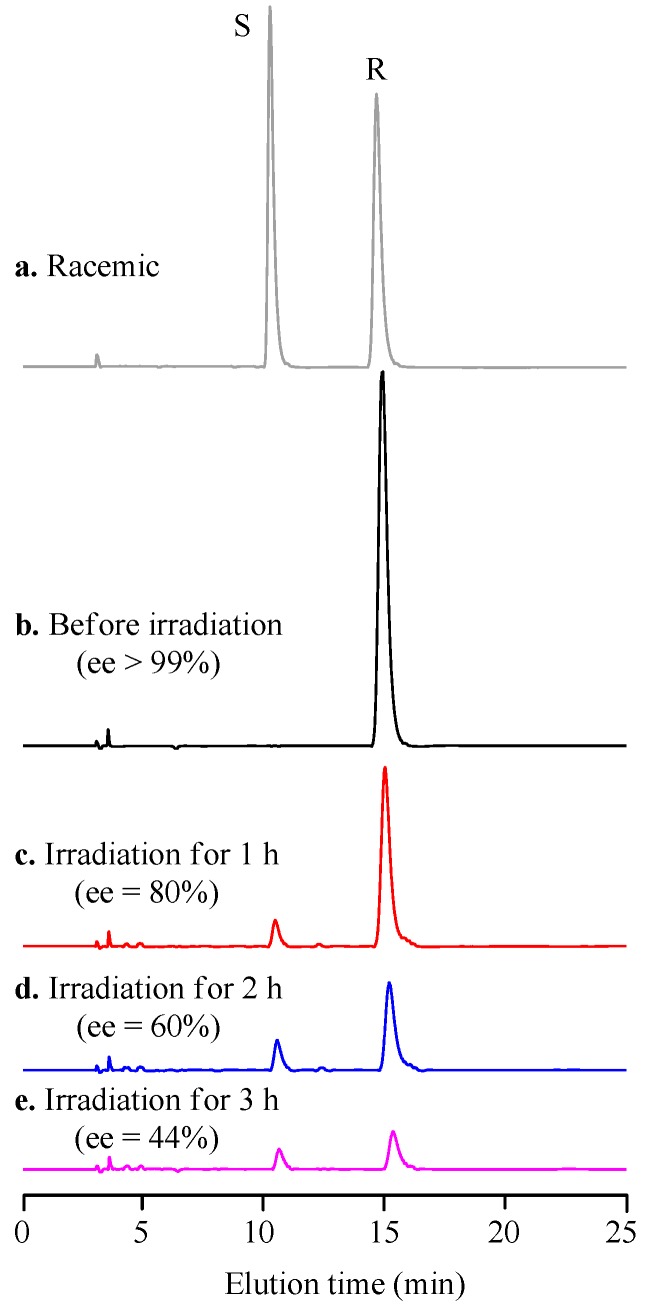
HPLC chromatograms of samples obtained from (*R*)-BINOL in a CH_3_CN solution upon irradiation ([(*R*)-BINOL]_o_ = 1.0 × 10^−3^ M, cell path = 5 mm, solution volume = ca. 1.75 mL) and standard samples: (**a**) standard racemic BINOL; (**b**) standard (*R*)-BINOL; (**c**–**e**) samples after irradiation for 1 h, 2 h, and 3 h, respectively.

In order to confirm the reason of the mutarotation, chromatographic resolution of the irradiated BINOL samples was performed (Figure 2). For resolution, a Daicel Chiralpak IA column based on amylose derivative was employed. Under the conditions in this work where the size of the column was 25 cm × 0.46 cm (i.d.), the flow rate was 0.5 mL/min, and the eluent was a mixture of hexane and dichloromethane (50/50, *v*/*v*), the racemic BINOL was completely resolved with chromatographic factors, k_1_ = 2.35 and α = 1.61 (Figure 2a). The sample before irradiation indicated only one signal which is ascribed to the (*R*)-isomer at an elution time of 14.9 min (Figure 2b) while the irradiated samples indicated the signals of the (*S*)-isomer at an elution time of 10.6 min and the relative intensity of this signal increased with irradiation time (Figure 2c–e). These observations unambiguously indicate that the decrease in CD intensity under irradiation is ascribed to racemization of BINOL. Photo racemization diaryl compounds having axial chirality including BINOL [29,30,31], a BINOL derivative [32], 1,1′-binaphthyl [33], a biphenyl derivative [34], and a 1,1′-biphenanthrene derivative [35,36] have been reported. In addition, photo-induced decomposition of one enantiomer BINOL in the presence of a protein is known [37].

The change in chirality due to irradiation was quantitatively analyzed by evaluating chirality in terms of Kuhn’s anisotropy factor, g_CD_, [38] which is defined as follows:

g_CD_ = 2(ε_L_ − ε_R_)/(ε_L_ + ε_R_)

where ε_L_ and ε_R_ are molar absorptivities of the system toward left- and right-handed circularly polarized light. Figure 1b,c indicate the plots of g_CD_ and enantiomeric excess (e.e.) against relative irradiation energy sum (relative absorbed dose) by the system at 223 nm and 318 nm. Since a decrease in UV intensity is observed in Figure 1a, “relative irradiation energy sum” (relative absorbed dose) [22] was chosen rather than irradiation time for the quantity on the horizontal axis in Figure 1b,c. It corresponds to the sum of irradiation time intervals (∆*t_i_*) used to change a CD spectrum to the directly following CD spectrum multiplied by UV absorbance (*Abs_i_*) corresponding to the former CD spectrum according to the following equation:

$$\mathrm{Relative}\;\mathrm{irradiation}\;\mathrm{energy}\;\mathrm{sum}=\sum_{i=0}^{n}\Delta t_{i}\times Abs_{i}.$$

Because g_CD_ is proportional to the enantiomeric excess (e.e.) of a compound, g_CD_ at each data point in Figure 1b,c can be correlated to the e.e. of BINOL on the basis of g_CD_ of optically pure (*R*)-BINOL before irradiation. Under the present experimental conditions where light intensity is constant, in other words, the number of photons per time hitting the system is constant throughout the reaction, the rate of decrease in g_CD_ and e.e. would depend only on and is proportional to the concentration of un-racemized (*R*)-BINOL (a first-order relation), which would lead to a concave upward curve with negative slopes increasing, with an increase in relative energy absorbed by the system. However, the relation’s g_CD_ and e.e. of BINOL with relative photon energy absorbed by the system at both 223 nm and 318 nm seem not to fit to a first-order relation. This would mean that the irradiated solution undergoes not only racemization but also another chemical transformation which affects the efficiency of photo racemization.

**Figure 3:**
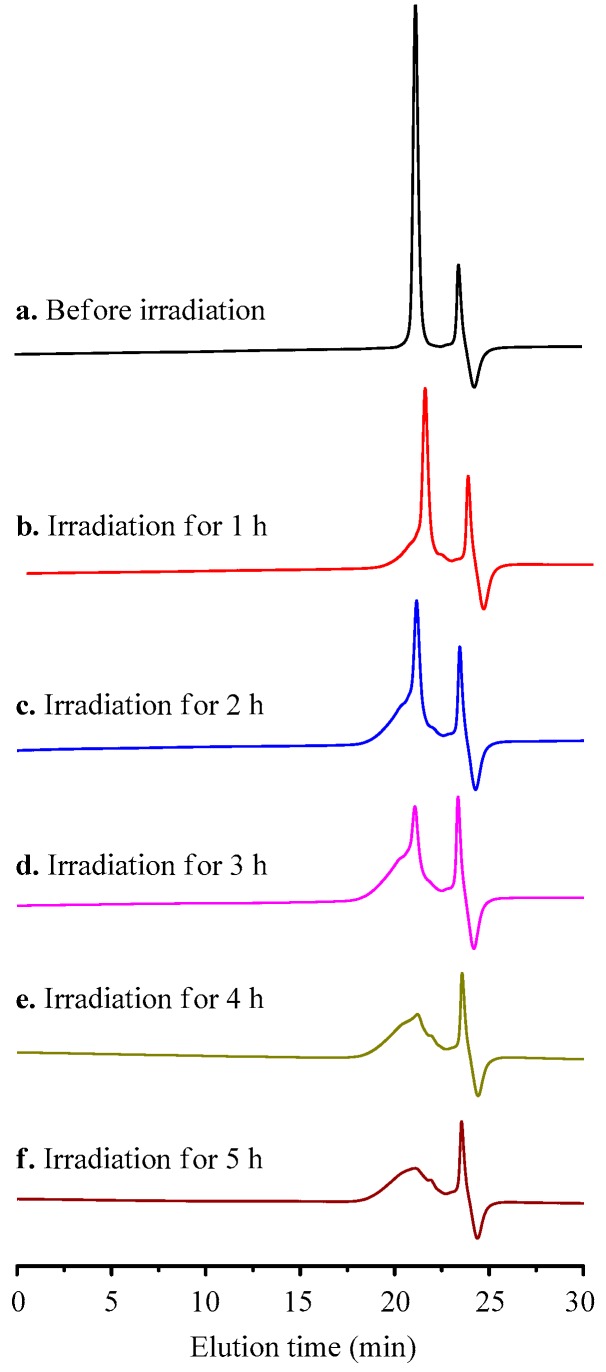
Size-exclusion chromatography (SEC) chromatograms of samples obtained from (*R*)-BINOL in a CH_3_CN solution upon irradiation ([(*R*)-BINOL]_o_ = 1.0 × 10^−3^ M, cell path = 5 mm, solution volume = ca. 1.75 mL) and a standard (*R*)-BINOL sample: (**a**) standard (*R*)-BINOL; (**b**–**f**) samples after irradiation for 1 h, 2 h, 3 h, 4 h, and 5 h, respectively.

About this aspect, information was obtained by size-exclusion chromatography (SEC) analyses of the products. Figure 3 shows SEC profiles of the samples before and after irradiation. The signal of BINOL appeared at around 21.2 min before irradiation, and additional signals emerged in the range of 17.6–22.6 min whose relative intensity increased with irradiation time. This observation clearly means that polymerization of BINOL takes place under irradiation. Although *M*_n_ of the product relative to standard polystyrenes is only less than a few hundred, the highest molar mass of the product corresponding to the rising point of the polymer peak at 17.6 min is ca. 2000. Thus, it is established that BINOL undergoes polymerization as well as racemization upon irradiation. The deviation of e.e.-vs.-relative energy plots in Figure 1 from a shape expected for a first-order reaction may be caused by the polymerization.

**Figure 4:**
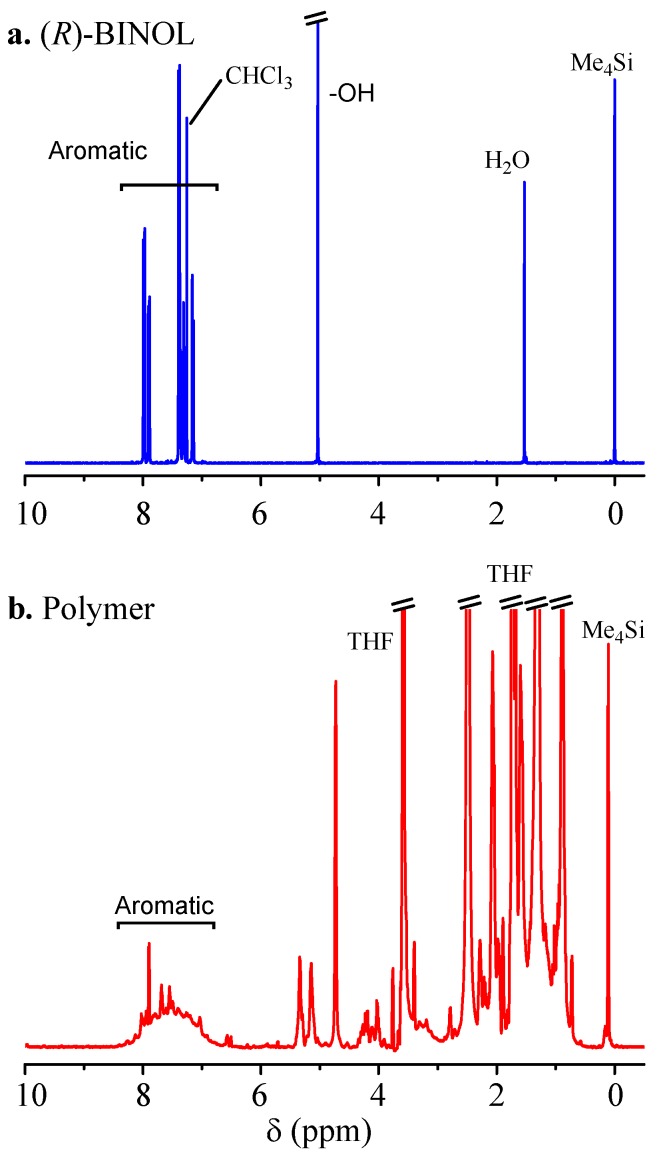
^1^H-NMR spectra: (**a**) (*R*)-BINOL taken in CDCl_3_; (**b**) the polymeric product obtained from (*R*)-BINOL in a CH_3_CN solution upon irradiation for 5 h ([(*R*)-BINOL]_o_ = 1.0 × 10^−^^3^ M, cell path = 5 mm) taken in THF-*d*_8_ [400 MHz, room temperature, Me_4_Si].

**Figure 5:**
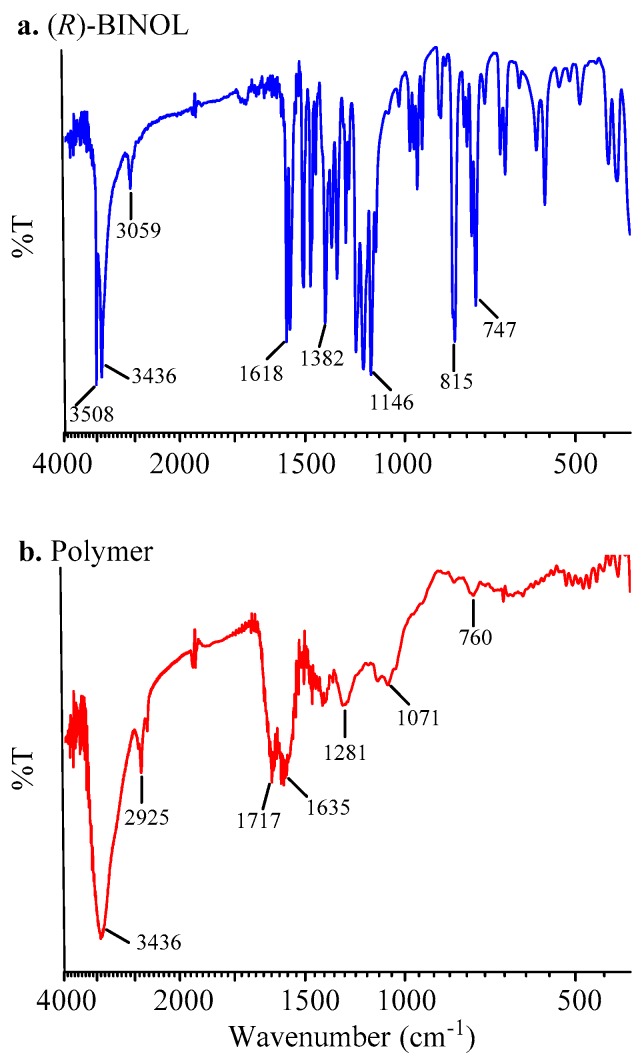
IR spectra: (**a**) (*R*)-BINOL; (**b**) the polymeric product obtained from (*R*)-BINOL in a CH_3_CN solution upon irradiation for 5 h ([(*R*)-BINOL]_o_ = 1.0 × 10^−^^3^ M, cell path = 5 mm) [KBr pellet].

The aforementioned decrease in UV spectral intensity of the BINOL solution in Figure 1a may also be ascribed to polymer formation. Although the structure of the polymerization product is not yet clear, the product seems to be almost optically inactive. Figure 4 and Figure 5 show the ^1^H-NMR and IR spectra, respectively, of the photo polymerization product along with those of (*R*)-BINOL. The broad aromatic signals in the NMR spectrum of the photo reaction product support that the product is a polymer (Figure 4). Further, it is noteworthy that rather intense signals were observed in the range of 0.8~5.33 ppm in addition to the aromatic signals. These results suggest that the polymeric product does not consist purely of BINOL units, but it may have a partially reduced structure and may have residues arising not only from BINOL but also from acetonitrile, the solvent. This aspect was further assessed by IR spectra (Figure 5). In the spectrum of the polymer, the signals were much broader than those of BINOL, which is consistent with the formation of a polymer. In addition, signals due to aromatic C–H out-of-plane bending vibration including the intense ones at 815 and 747 cm^−1^ are much weaker in the polymer spectrum which supports the partial loss of aromaticity due to reduction. Further, the polymer spectrum indicated rather intense signals at around 1700 cm^−1^ which would not be observed in a polymer consisting only of BINOL units and may indicate the presence of C=N or C=O functions, suggesting that acetonitrile might have a role in the photo polymerization or that C=O species that have been proposed to form upon irradiation [30,31] might be incorporated into the polymer chain. It is also noteworthy that, in the IR spectra, the signals in the range of 3000–4000 cm^−1^ were largely broadened through irradiation. This might entail the formation of intra-molecular hydrogen-bonding between –OH groups of neighboring units in the polymeric product with various strengths and forms.

Elemental analysis of a polymer prepared in a repeated experiment under conditions similar to those for the HPLC and SEC experiments with an irradiation time of 4 h further supported the conclusions obtained from the NMR and IR spectra. While the ideal ratio of elements for a polymer comprising purely of BINOL units may be close to the value for BINOL (C, 83.90%; H, 4.93%), the experimental results on the polymer was C, 58.95%; H, 4.73%; N, 3.54%. These results support that the product has N atoms arising from CH_3_CN and that BINOL was partially reduced on the incorporation into the polymer chain. Although the exact structure of the polymer is not yet known and the reaction mechanism leading to the polymer is not yet clear at this point, studies are under way to shed light on these aspects.

**Figure 6:**
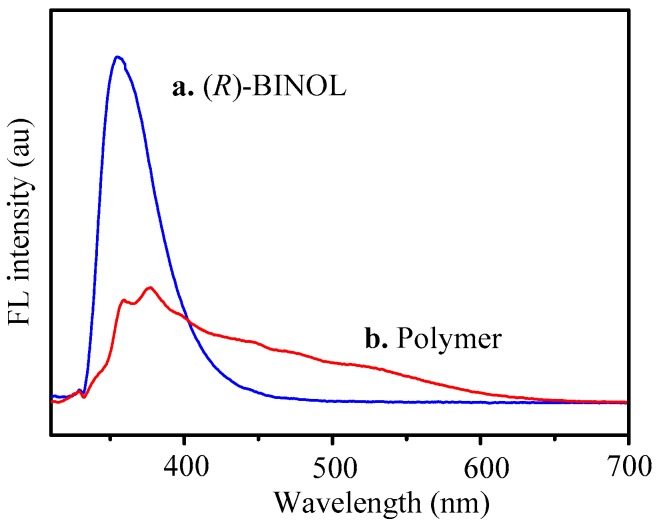
Emission spectra: (**a**) (*R*)-BINOL in a CH_3_CN solution; (**b**) the polymeric product obtained from (*R*)-BINOL in a CH_3_CN solution upon irradiation for 4.5 min ([(*R*)-BINOL]_o_ = 1.0 × 10^−^^6^ M, cell path = 10 mm) [λ_ex_ 300 nm]. Intensity was normalized to a constant absorbance at 300 nm.

Emission spectra of BINOL and the polymeric product obtained by irradiation are shown in Figure 6. While (*R*)-BINOL indicated a rather sharp emission peak at around 355 nm, the polymeric product showed much broader emission signals. Emission efficiencies of BINOL and the polymer were 0.04 and 0.03, respectively, as determined using 9,10-diphenylanthracene (0.90 in cyclohexane) as standard [39]. The value for BINOL well matches the one in the literature [40]. The broader signals of the polymer may mean that the polymer has an extended π-conjugation system derived from the BINOL backbone. Changes in emission spectra of BINOL and related compounds due to their photo degradation have been reported [31,41,42]; however, polymer formation during the photo reactions have never been proposed.

In the context of polymer formation, a question may be which occurs faster, racemization or polymerization. Although exact kinetic comparisons between the two have not been conducted in this work, Figure 1b,c indicate that the decrease in g_CD_ is slower than expected from a first-order reaction. In a first-order reaction, the drop in g_CD_ with an increase in relative irradiation energy should be sharper. This may mean that polymerization of BINOL can occur before it is racemized and that a unit in a polymer derived from BINOL loses its chirality afterwards due to in-chain racemization and/or reduction.

In addition, it was found that the photo racemization and polymerization was faster at a lower concentration. The reactions at 1.0 × 10^−3^ M (Figure 2, Figure 3, Figure 4 and Figure 5) and at 1.0 × 10^−4^ M (Figure 1) were completed roughly within 5 h and 15 min, respectively, using the same irradiation set-up. This might mean that inter-molecular interactions have a role in the reaction mechanism.

Further, whether TCT upon irradiation is feasible from the view of rotation barrier of BINOL is an important point in discussing the reaction mechanism in this study. Rotation barriers of BINOL have been assessed by thermal racemization experiments and theoretical calculations; the experimental barriers in naphthalene and diphenyl ether were 155.5 kJ/mol and 158 kJ/mol, respectively, and theoretically predicted barriers were in the range of 158.3–249.4 kJ/mol and varied depending on the transition-state conformation [43]. In the absorbance spectrum of BINOL (Figure 1a), the lowest-energy absorbance edge is at around 350 nm, which corresponds to 342 kJ/mol. This means that even the lowest-energy photon absorbed by BINOL has a high enough energy to induce TCT.

## 3. Conclusions

(*R*)-BINOL was found to racemize and polymerize upon photo irradiation. The racemization may occur through TCT of BINOL through photo excitation. Although TCT has been reported for biphenyl through theoretical calculations [23] and has been experimentally established for aromatic–aromatic junctions in polymers leading to a helical structure upon irradiation using CPL [20,21,22,44], reports upon photo racemization of axially chiral compounds are rare. The finding of co-occurring photo racemization and polymerization of optically active BINOL is unprecedented, while there have been only a few related publications about the photo-induced racemization of diaryl compounds [29,30,31,32,33,34,35,36]. The observations in this work are in sharp contrast to the general recognition that BINOL is a rather robust chiral backbone to be used for catalysis and for the construction of chiral materials. Studies are under way to clarify the polymer structure and the mechanism of the photo racemization and polymerization as well as to find a way to make an optically active polymer from racemic BINOL with the aid of light.

## Appendix A

(*R*)- and racemic BINOL (Wako Pure Chemical Industries, Ltd., Japan) and acetonitrile (Kanto Chemical Co., Inc., Japan) were used as purchased. Irradiation was conducted using an Ushio Optical Modulex SXUID500MAMQQ 500-W Hg–Xe lamp under a N_2_ atmosphere at ambient temperature (ca. 23 °C). NMR spectra were recorded on a JEOL ESC400 spectrometer (400 MHz for ^1^H, 100 MHz for ^13^C). Size-exclusion chromatography (SEC) measurements were carried out using a chromatographic system consisting of a JASCO DG-980-50 degasser, a HITACHI L-7100 pump, a HITACHI L-7420 UV-Vis detector, and a HITACHI L-7490 RI detector, equipped with TOSOH TSKgel G3000H_HR_ and G6000H_HR_ columns (30 × 0.72 (i.d.) cm) connected in series (eluent: THF, flow rate: 1.0 mL/min). HPLC resolution was conducted using a chromatographic system consisting of a JASCO DG-980-50 degasser, a JASCO PU-980 pump, and a JASCO UV-2070plus UV detector (230 nm) equipped with a Daicel Chiral Pak IA column (25 cm × 0.46 cm (i.d.)) with a mixture of hexane and dichloromethane (50/50, *v*/*v*) as eluent. Circular dichroism (CD) spectra were taken with a JASCO-820 spectrometer. UV-Vis spectra were measured on JASCO V-550 and V-570 spectrophotometers. FT-IR spectra were recorded on a JASCO FT/IR-6100 spectrometer. Emission spectra were taken using a JASCO FP-8500 fluorescence spectrophotometer.
